# Spectral Ranking of Causal Influence in Complex Systems

**DOI:** 10.3390/e23030369

**Published:** 2021-03-20

**Authors:** Errol Zalmijn, Tom Heskes, Tom Claassen

**Affiliations:** 1 Institute for Computing and Information Sciences, Radboud University, 6525 EC Nijmegen, The Netherlands; Tom.Heskes@ru.nl (T.H.); tomc@cs.ru.nl (T.C.); 2 ASML Research Department, 5504 DT Veldhoven, The Netherlands

**Keywords:** complex systems, time series, transfer entropy, eigenvector centrality, original information, node importance, coupled Lorenz systems

## Abstract

Similar to natural complex systems, such as the Earth’s climate or a living cell, semiconductor lithography systems are characterized by nonlinear dynamics across more than a dozen orders of magnitude in space and time. Thousands of sensors measure relevant process variables at appropriate sampling rates, to provide time series as primary sources for system diagnostics. However, high-dimensionality, non-linearity and non-stationarity of the data are major challenges to efficiently, yet accurately, diagnose rare or new system issues by merely using model-based approaches. To reliably narrow down the causal search space, we validate a ranking algorithm that applies transfer entropy for bivariate interaction analysis of a system’s multivariate time series to obtain a weighted directed graph, and graph eigenvector centrality to identify the system’s most important sources of original information or causal influence. The results suggest that this approach robustly identifies the true drivers or causes of a complex system’s deviant behavior, even when its reconstructed information transfer network includes redundant edges.

## 1. Introduction

Semiconductor lithography systems are extremely complicated electromechanical systems, capable of sub-nanometer positioning and sub-milliKelvin temperature control, while generating extreme ultraviolet light from laser-pulsed tin plasma. It is notoriously difficult to fully understand the complex interactions among thousands of observed variables affecting the output of such systems. Model-based approaches alone are inadequate to effectively diagnose rare or new system issues, as they inherently do not model abnormal behavior. To efficiently, yet reliably, reduce the search space of potential causes, we consider both local and global causal influences of a complex system’s components, as measured by Schreiber’s transfer entropy [1] and Bonacich’s eigenvector centrality [2], respectively. Transfer entropy quantifies predictive information transfer as a potential signature of causality between two stationary time series, providing direction, strength and delay of linear or non-linear interactions. For time series with a Gaussian distribution, transfer entropy reduces to Granger causality [3]. However, being a bivariate measure, transfer entropy naturally disregards the multivariate nature of interactions in complex systems. Therefore, multivariate approaches use conditional transfer entropy [4] to separate true cause–effect relations from mere correlations, i.e., direct from so-called transitive indirect or semi-metric, and thus redundant relations, by iteratively conditioning out (subsets of) all other time series. Unfortunately, such an information decomposition is infeasible in (real time) diagnosis or prognosis of high-dimensional technological complex systems, due to its exponential scaling of computational costs with time-series dimension. Instead, we use a ranking algorithm that relies on standard transfer entropy in exhaustive, but computationally feasible bivariate interaction analysis of a complex system’s multivariate time series, resulting in an information transfer network that is likely to contain redundant edges. However, Benzi et al. [5] show that eigenvector centrality represents the upper limit of Katz centrality [6], where the global influence of nodes is essentially determined by the longest possible paths in the network, which implies its insensitivity to shortest paths, including transitive indirect or semi-metric, and thus redundant edges. In addition, empirical studies by Kalavri et al. [7] revealed that PageRank (or eigenvector centrality) yields similar rankings when computed with, or without, a graph’s (first-order) semi-metric edges. For diagnostic purposes as envisioned, we suffice to apply standard transfer entropy for approximative network inference of a complex system’s true causal structure and then use graph eigenvector centrality for computationally efficient, yet consistently accurate ranking of probable causes or key drivers of the system under disturbance.

This study relates most closely to the work of Streicher et al. [8], who implemented this approach into a two-step ranking algorithm for (chemical) plant-wide fault detection and diagnosis.

In this paper, we introduce the measures of transfer entropy and eigenvector centrality and describe the ranking algorithm as used. In rolling window analysis of a simulated time series representing two bidirectionally coupled Lorenz systems, we compare its causal network inference results to those of a constraint-based algorithm for multivariate causal analysis. The rolling analysis approach also allows the ranking algorithm to estimate the global influence of each state variable as exerted on the coupled Lorenz systems over time. Finally, we assess the ranking algorithm in diagnosing a real-world industrial system issue, using a higher-dimensional time series.

## 2. Applied Algorithms and Methods

### 2.1. Transfer Entropy

Transfer entropy (TE) is an information-theoretic implementation of Wiener’s notion of causality applied to time series [9], whereby the cause precedes—and contains unique information about—the effect. Consider two stationary ergodic Markov processes *X* and *Y* (or $X^{\left(i\right)}$ and $X^{\left(j\right)}$ as below) and their corresponding time series $\left\{x_{1},x_{2},...,x_{M}\right\}$ and $\left\{y_{1},y_{2},...,y_{M}\right\}$ of *M* samples. Transfer entropy quantifies reduction in uncertainty about future states of a source process *X*, when passed states of a target process *Y* are observed in addition to passed states of *X* itself. As an asymmetric measure based on transition probabilities, transfer entropy naturally incorporates directional and dynamic information, which may imply causation between *X* and *Y*:

(1) $$\begin{array}{cc} TE_{X\to Y}^{\left(k,l\right)}\left(t,\tau \right) & =\sum_{y_{t},y_{t-1}^{\left(l\right)},x_{t-\tau }^{\left(k\right)}}p\left(y_{t},y_{t-1}^{\left(l\right)},x_{t-\tau }^{\left(k\right)}\right)\log_{b}\frac{p(y_{t}|y_{t-1}^{\left(l\right)},x_{t-\tau }^{\left(k\right)})}{p(y_{t}|y_{t-1}^{\left(l\right)})} \end{array}$$

where $x_{t}$ and $y_{t}$ represent states of *X* and *Y* at time *t*, while $TE_{X\to Y}^{\left(k,l\right)}\left(t,\tau \right)$ indicates maximized information transfer from *X* to *Y*, computed across a range $D=\{0,1,2,...,\tau_{max}\}$ of embedding delays τ, such that $\max_{\tau \epsilon D}\left\{TE_{X\to Y}^{\left(k,l\right)}\left(t,\tau \right)\right\}=TE_{X\to Y}^{\left(k,l\right)}\left(t,\;\underset{\tau \epsilon D}{arg max}\left\{TE_{X\to Y}^{\left(k,l\right)}\left(t,\tau \right)\right\}\right)$. The embedding dimensions *k* and *l* denote the number of passed states in *X* and *Y* used to condition the probabilities of transition to the next state of *Y* (or *X*) represented by $x_{t-\tau }^{\left(k\right)}=\left\{x_{t-\tau -k+1},x_{t-\tau -k+2},...,x_{t-\tau }\right\}$ and $y_{t-1}^{\left(l\right)}=\left\{y_{t-1-l+1},y_{t-1-l+2},...,y_{t-1}\right\}$. The logarithm base b=2 defines the informational unit of transfer entropy in bits, i.e., reduction in average code-length required to optimally encode the target variable (effect), given passed states of the source variable (cause) and target variable. Herein, we keep the embedding dimensions at the commonly used k=l=1, mostly for computational reasons. For every pair $\left(X^{\left(i\right)},X^{\left(j\right)}\right)$ from a multivariate time series $\left\{X^{\left(1\right)},X^{\left(2\right)},...,X^{\left(N\right)}\right\}$, we apply Equation (1) to estimate information transfer TE and thereby determine the candidate source times series $X_{\left(d\right)}$ and target time series $X_{\left(r\right)}$. We then generate 1/(α=0.05)−1 surrogates $\left\{X_{\left(d\right)}^{{}^{\prime }\left(1\right)},X_{\left(d\right)}^{{}^{\prime }\left(2\right)},...,X_{\left(d\right)}^{{}^{\prime }\left(19\right)}\right\}$, which share their amplitude distribution and power spectrum with original time series $X_{\left(d\right)}$, using the iterative amplitude adjusted Fourier transform (iAAFT) proposed by Schreiber et al. [10]. Following this study, we estimate information transfer $TE^{{}^{\prime }}$ from each surrogate to (original) target time series $X_{\left(r\right)}$ and obtain $\left\{TE_{1}^{{}^{\prime }},...,TE_{19}^{{}^{\prime }}\right\}$. If $TE>max\{TE_{1}^{{}^{\prime }},...,TE_{19}^{{}^{\prime }}\}$, information transfer TE is considered to be significant or non-significant otherwise. The resultant information transfer network, given by a directed weighted graph G=(V,E), comprises a set $V={\left\{v_{i}\right\}}_{i=1}^{N}$ of *N* nodes and $E=\{e_{ij}=\;\left(v_{i},v_{j}\right)\}$ of edges. Each edge $e_{ij}$ connects a source node $v_{i}$ to target node $v_{j}$ with strength $w_{ij}$, i.e., information transfer TE.

### 2.2. Eigenvector Centrality

To diagnose performance issues or even failures within technological complex systems, we wish to locate where perturbations enter the system and propagate, causing downstream effects throughout the system. Therefore, we consider an information transfer network wherein the (main) sources of original information can be identified by measurement and ranking of each node’s global network influence termed centrality or importance. Centrality, the basic principle of Google’s search engine [11], has proven useful to measure and rank a page’s relevance based on its inbound links. Here, we use out-degree eigenvector centrality, which defines the centrality $c(v_{i})$ of a node $v_{i}\;\epsilon \;V=\left\{v_{1},...,v_{n}\right\}$ as proportional to the summed centralities of its outbound neighbors:

(2) $$\begin{array}{cc} c\left(v_{i}\right)=\lambda^{-1}\sum_{j=1}^{N}W_{ij}c\left(v_{j}\right) & ,\;\mathrm{or}\;Wc=\lambda c \end{array}$$

where $\lambda^{-1}$ is a proportionality factor and *c* is the eigenvector of centralities associated with eigenvalue λ of adjacency matrix *W*, whose entries $w_{ij}$ denote information transfer from node $v_{i}$ to node $v_{j}$ in graph *G*. If *W* is non-negative and irreducible, the Perron-Frobenius theorem [12,13] ensures there is a unique vector $c_{1}$ of *N* centralities $c_{1}\left(v_{i}\right)>0\;,\forall \;v_{i}$ associated with the largest positive eigenvalue $\lambda_{1}=\rho \left(W\right)$ or spectral radius of *W*, satisfying Equation (2). Usually $c_{1}$ is normalized, such that each entry indicates the centrality or importance of a node $v_{i}$ in graph *G* on a relative scale from 0 to 1. Alternatively, matrix *W* is normalized to a transition matrix *P*, whose entries $p_{ij}$ denote probabilities of transition from node $v_{i}$ to node $v_{j}$ in a random walk on graph *G* or Markov chain while $\sum_{j=1}^{N}p_{ij}=1$, such that:

(3) $$\begin{array}{c} p_{ij}=\left\{\begin{array}{cc} w_{ij}/\sum_{j=1}^{N}w_{ij}, & \;\;\mathrm{if}\;\;\sum_{j=1}^{N}w_{ij}\neq 0\\ 0, & \;otherwise \end{array}\right. \end{array}$$

Following Google’s PageRank approach [14], we modify matrix *P* by adding a teleportation probability γϵ〈0,1〉 and an all-ones matrix *J* to obtain an N×N, irreducible, positive matrix $P^{{}^{\prime }}$:

(4) $$\begin{array}{c} P_{\gamma }^{{}^{\prime }}=\gamma P+\frac{1}{N}\left(1-\gamma \right)J \end{array}$$

where a random walker at node $v_{i}$ follows an edge with probability γ or jumps to any other node in the *N*-nodes network with probability (1−γ). Herein, we use PageRank’s typical value of γ=0.85. Considering the Markov chain associated with matrix $P_{\gamma }^{{}^{\prime }}$, the Perron–Frobenius theorem ensures an unique stationary probability distribution that matches the eigenvector ${\vec{\pi }}_{1}$ of $P_{\gamma }^{{}^{\prime }}$ associated with eigenvalue $\lambda_{1}\left(P_{\gamma }^{{}^{\prime }}\right)=1$, such that ${\vec{\pi }}_{1}=P_{\gamma }^{{}^{\prime }}{\vec{\pi }}_{1}$. Eigenvector ${\vec{\pi }}_{1}$ is usually computed via power iteration [15] in *k* steps: ${\vec{\pi }}_{1}=\lim_{k\to \infty }{\vec{\pi }}^{\left(k\right)}=\lim_{k\to \infty }P_{\gamma }^{{}^{\prime }\left(k\right)}{\vec{\pi }}^{\left(0\right)}$, where ${\vec{\pi }}^{\left(0\right)}$ denotes an initial distribution.

We use the FaultMap algorithm (https://github.com/SimonStreicher/FaultMap, accessed on 20 May 2015) from Streicher [8], which, to our knowledge, is the only open-source implementation of the method as described above and summarized in the pseudo-code of Algorithm 1, as shown below.

**Algorithm 1** FaultMap.Information Transfer Network Inference
1:**Input:***N*-dimensional time series $\left\{X^{\left(1\right)},X^{\left(2\right)},...,X^{\left(N\right)}\right\}$, of *M* samples from system *X* represented by: $x^{\left(i\right)}=\left\{x_{1}^{\left(i\right)},x_{2}^{\left(i\right)}...,x_{M}^{\left(i\right)}\right\}\;\epsilon \;R^{M}$, statistical significance threshold δ in, e.g., a rank-order test using iAAFT surrogates, embedding delay range $\tau_{max}$ and embedding history lengths *k* and *l* for time series *i* and time series j(≠i)2:**Output:** adjacency matrix $W\;\epsilon \;R^{N\times N}$, where entry (i,j) represents information transfer from node *i* to node *j*3:**for**i←1 to *N* **do**4:    **for** j←1 to N,j≠i **do**5:        **for** τ←0 to $\tau_{max}$ **do**6:           compute $TE_{i\to j}\left(k,l,\tau \right)$ by Equation (1)7:           **if** $TE_{i\to j}=0<\delta <\max_{0⩽\tau ⩽\tau_{max}}\left\{Equation\left(1\right)\right\}$ **then**8:               $W_{ij}\leftarrow TE_{i\to j}$9:           **else**10:               $W_{ij}\leftarrow 0$11:           **end if**12:        **end for**13:    **end for**14:**end for**
Spectral Centrality Ranking
1:**Input:** matrix $P_{\gamma }^{{}^{\prime }}=\gamma P+\frac{1}{N}\left(1-\gamma \right)J$ where γϵ〈0,1], ranking distance ϵ2:**Output:** node centrality score vector $\pi_{1}$3:initialize $\pi^{\left(0\right)}$ with probabilities [(1/N,1/N,...,1/N)]4:**while**$|\pi^{\left(k+1\right)}-\pi^{\left(k\right)}|>\epsilon$**do**5:    compute eigenvector $\pi^{\left(k+1\right)}$ of matrix $P_{\gamma }^{{}^{\prime }}$ associated    with eigenvalue $\lambda_{1}\left(P_{\gamma }^{{}^{\prime }}\right)=1$, such that $\pi_{1}=P_{\gamma }^{{}^{\prime }}\pi_{1}$6:**end while**


### 2.3. Validation

In what follows, we assess the method’s accuracy in causal inference and node ranking using simulated time series, followed by root cause analysis from real-world diagnostic data. Firstly, we consider a system $S_{\left(L_{1}⇄L_{2}\right)}$ of two bidirectionally coupled Lorenz systems $L_{1}$ and $L_{2}$, investigated by Wibral et al. [16] and given by:

(5a)X˙1(t)=10.0(Y1(t)−X1(t))(5b)Y˙1(t)=X1(t)(25.0−Z1(t))−Y1(t)+0.1Y22(t−3)(5c)Z˙1(t)=X1(t)Y1(t)−2.67Z1(t)(5d)X˙2(t)=10.0(Y2(t)−X2(t))(5e)Y˙2(t)=X2(t)(28.0−Z2(t))−Y2(t)+0.05Y12(t−5)(5f)Z˙2(t)=X2(t)Y2(t)−2.67Z2(t)

The bidirectional coupling $Y_{1}⇄Y_{2}$ is governed by time-delayed quadratic terms. We used Pydelay [17] to generate a multivariate time series $\left\{X_{1},X_{2},Y_{1},Y_{2},Z_{1},Z_{2}\right\}$ of 150K samples by numerically integrating Equations (5a)–(5f) with step size dt=0.01 and initial conditions $X_{1}\left(0\right)=X_{2}\left(0\right)=1.0$, $Y_{1}\left(0\right)=Y_{2}\left(0\right)=0.97$ and $Z_{1}\left(0\right)=Z_{2}\left(0\right)=0.99$. To assess FaultMap’s accuracy in causal network reconstruction against other model-free approaches, we use PCMCI (v4.0) from Runge [18] as a distinct, constraint-based, multivariate alternative. FaultMap estimates edge-weight $w_{ij}$ as $\Delta TE\left(\tau \right)=TE_{X^{\left(i\right)}\to X^{\left(j\right)}}\left(\tau \right)-TE_{X^{\left(j\right)}\to X^{\left(i\right)}}\left(\tau \right)$ using the Kraskov–Stögbauer–Grassberger estimator for transfer entropy in the Java Information Dynamics Toolkit from Lizier [19]. PCMCI performs condition selection at an optimized significance level α using Akaike’s information criterion, followed by a conditional independence test where we use the linear ParCorr test (at α=0.05) instead of the computationally expensive nonlinear CMI test. Following the recommended sample size of Bauer [20], we use a rolling analysis window $W_{s}$ of 2K samples to define 50 adjacent time series slices for causal network reconstruction of the coupled Lorenz systems by both algorithms and node importance ranking by FaultMap only.

## 3. Results and Discussion

### 3.1. Coupled Lorenz Systems

**Figure 1:**
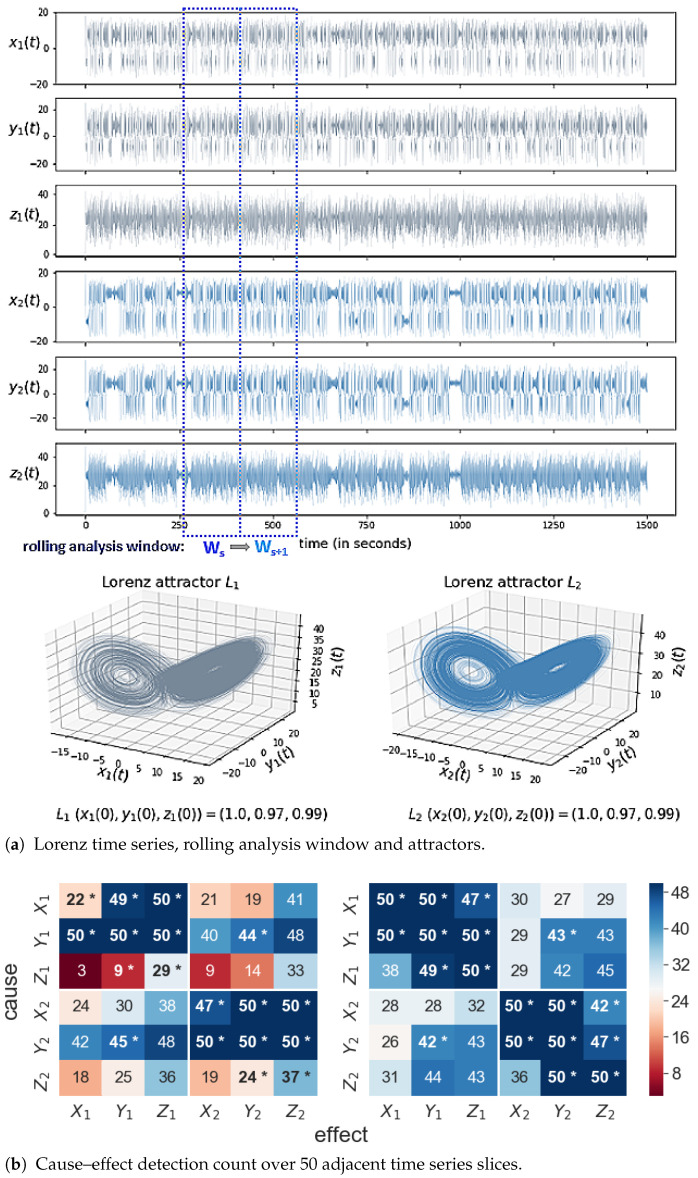
(**a**) Time series of system $S_{\left(L_{1}⇄L_{2}\right)}$ composed of bidirectionally delay-coupled Lorenz systems $L_{1}$ and $L_{2}$, generated from Equations (5a)–(5f). (**b**) Heatmaps of detection count per cause–effect relation, for FaultMap (left) and PCMCI (right). Direct cause–effect relations are denoted by (*).

**Figure 2:**
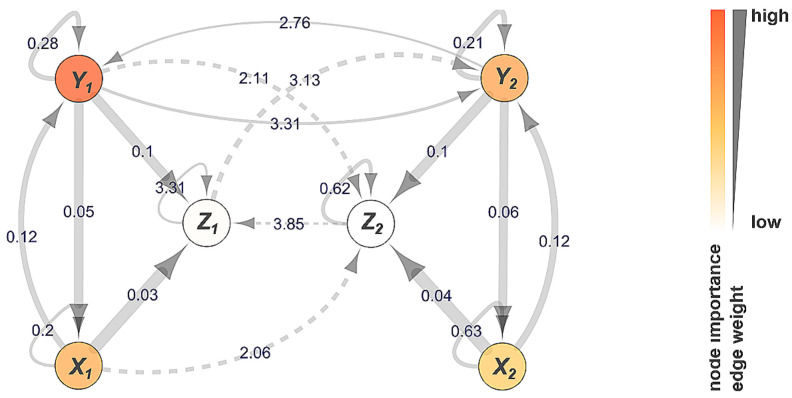
Information transfer network of two bidirectionally delay-coupled Lorenz systems. Edges indicate direct (⟶) or transitive indirect (⤏) information transfer. Edge annotations denote information transfer delay (sec). Node importance indicates a node’s global network influence. Edge-weight represents level of information transfer ($w_{ij}$).

**Figure 3:**
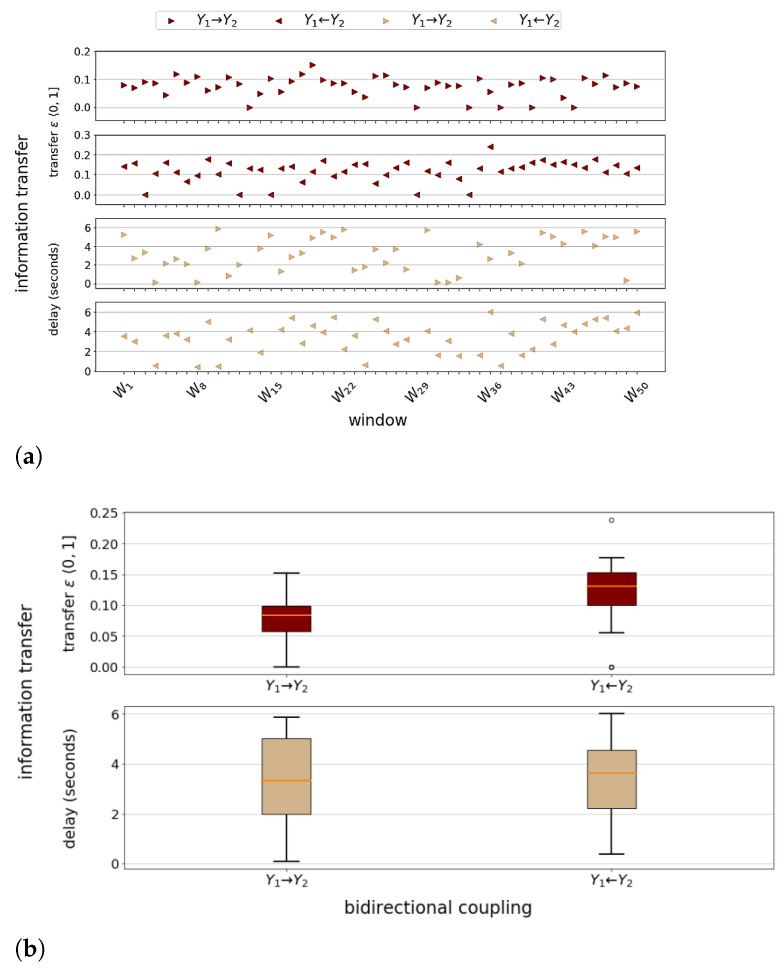

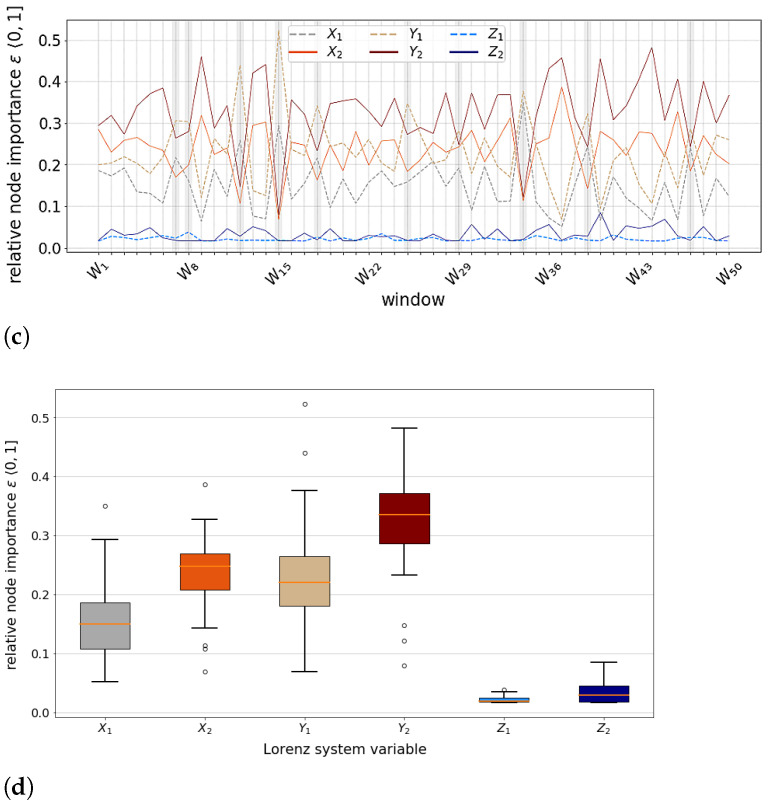
Dynamic information transfer via bidirectional delay-coupling $Y_{1}⇄Y_{2}$ of Lorenz systems $L_{1}$ and $L_{2}$, and dynamic importance of the Lorenz system state variables. (**a**) Dynamic information transfer (delay) via bidirectional delay-coupling $Y_{1}⇄Y_{2}$ between Lorenz systems $L_{1}$ and $L_{2}$. (**b**) Distribution of information transfer (delay) in 3a. (**c**) Dynamic importance of state variables in delay-coupled Lorenz systems $L_{1}$ and $L_{2}$. (**d**) Distributions of Lorenz system state variable importance in 3c.

Figure 1 depicts the rolling window analysis approach, the butterfly-shaped attractors of the coupled Lorenz systems in phase space (x,y,z) and cause–effect detection count heatmaps for both algorithms. Figure 2 exemplifies FaultMap’s rolling window analysis results of which Figure 3 specifically reports information transfer (delay) via coupling $Y_{1}⇄Y_{2}$ and global influence of *X*-, *Y*- and *Z*-variables on the coupled Lorenz systems. The heatmaps in Figure 1b show the total count of each cause–effect relation detected by FaultMap or PCMCI over 50 adjacent time series slices. Firstly, PCMCI’s linear ParCorr test detected nonlinear coupling $Y_{1}⇄Y_{2}$ remarkably well, with a rate of 85% vs. 89% by FaultMap and achieved a notably higher detection rate (95%) of (direct) causal links throughout the coupled Lorenz systems than FaultMap (83%). ParCorr’s sensitivity to nonlinear interactions has been previously discussed by Krich et al. [21]. PCMCI’s higher detection rate of direct links compared to FaultMap is mainly due to its 100% detection rate of self-influence at each system state variable, whereas FaultMap only reached a 100% detection rate of self-influence at the coupled system state variables $Y_{1}$ and $Y_{2}$. We could not find an obvious explanation for the algorithms’ differing detection rates of self-influence (see the heatmaps’ diagonal), but may argue that self-influence as subset of the previously defined edge-set *E*, or formally $\{e_{ii}=\left(v_{i},v_{i}\right)\}\subseteq E$, is irrelevant in the context of Equation (2). Lastly, note that unlike what we would expect from its multivariate causal network reconstruction designed to distinguish direct from indirect effects, PCMCI detected 23% more transitive indirect links than FaultMap.

Due to the transitivity of bivariate information transfer, most (if not all) networks inferred by FaultMap include direct and indirect connections, all of which passed strict significance tests, as $Y_{1}\to Y_{2}\to Z_{2}$ and $Y_{1}⤏Z_{2}$ shown in Figure 2. The coupled Lorenz systems are easily discernible by two subnetworks $\left(X_{1},Y_{1},Z_{1}\right)$ and $\left(X_{2},Y_{2},Z_{2}\right)$ that exhibit distinct levels of information transfer with similar time delays in the range of milliseconds. At a rate of at least 98%, FaultMap detected the same edges X→Y, Y→X, X→Z and Y→Z indicating (normalized) net influence within both Lorenz subsystems, as Gencaga et al. [22] found for a single Lorenz system. PCMCI shows similar detection results for these edges (see heatmaps). It is important to note that the rolling window approach took PCMCI 495 min of runtime on a 24-core HPC node with 192 Gb for causal analysis vs. 58 min by FaultMap for a two-step causal analysis and node ranking. PCMCI’s considerably higher computational costs compared to FaultMap obviously limit its applicability for the diagnostic purposes considered above.

In Figure 3a,b, we focus on reconstruction of time delays in coupling Y1⇄Y2, given the modeled delays in Equations (5b) and (5e). Figure 3a reveals the dynamic nature of bidirectional information transfer in terms of strength and delay. The median difference (≈0.05) of information transfer distributions in Figure 3b suggests that Lorenz system $L_{2}$ predominantly drives $L_{1}$, which is reasonable to expect since the coupling strength (0.1) in Equation (5b) is twice the coupling strength (0.05) in Equation (5e). Given coupling delays of 3 and 5 s, the distribution of reconstructed interaction delays seems realistic but may be impacted by lag synchronisation of the Lorenz systems, as suggested by Coufal et al. [23]. Figure 3c shows highly dynamic global influence of particularly *X*- and *Y*-variables, while the *Y*-variables remain the driving force within their respective Lorenz subsystem at all times. Grey-colored bars highlight time windows in which $L_{1}$ is identified as driving, and $L_{2}$ as driven subsystem. Since the coupling strengths are constant, $Y_{1}\to Y_{2}$ is likely to dominate $Y_{2}\to Y_{1}$ in strength when the $Y_{2}$ state reaches vanishingly low values relative to the $Y_{1}$ state. The median difference across all node importance distributions in Figure 3d reflects the aforementioned drive–response relations in, and between, the interacting Lorenz systems. It might explain FaultMap’s 100% detection rate of *Y*-variable self-loops vs. lower rates of all other self-loops. The outcome of both *Y*-variables as Lorenz system key driver complies with the Lorenz model of Rayleigh–Bénard convection [24], where temperature difference drives convective heat transfer in addition to conduction at Rayleigh number Ra≥25 (see Equations (5b) and (5e)). To our knowledge, this is the first rolling window analysis to date, capturing the dynamics of time-varying information transfer or global influence (importance) of state variables within interacting Lorenz systems. Our findings may enable automated identification of monitoring observables for performance (anomaly) diagnostics or predictive maintenance within technological complex systems. The ability to capture time-varying importance of a complex system’s state variables is also relevant in time-series analysis of natural complex systems, including the Earth’s climate.

### 3.2. Technological Complex Systems

**Figure 4:**
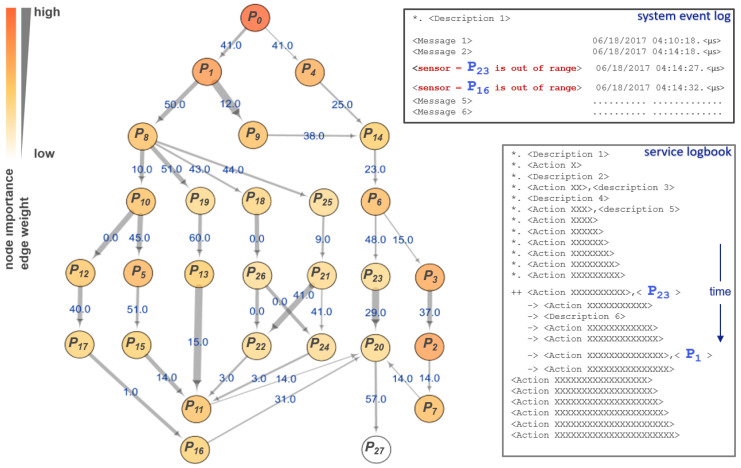
Top-ranked node $P_{0}$ (root cause) transfers original information towards event $P_{27}$ via a network of collateral effects $\left\{P_{1},...,P_{26}\right\}$ within an ASML subsystem. (Figure 2 legend applies here.)

Nanolithography systems are among the most complex technological systems today, capable of sub-nanometer positioning and sub-milliKelvin temperature control, even as system modules accelerate at up to 15 Gs. Such systems are particularly challenging for model-based diagnosis of rare or new issues, due to nonlinear interactions across multiple time and spatial scales. To assess FaultMap’s potential in diagnosis of issues within such systems, we investigate temperature, flow and pressure instability within an ASML subsystem. Therefore, we use a multivariate time series of 315 binary samples from 366 parameters related to the problem. As shown in Figure 4, FaultMap identified parameter $P_{0}$ as a primary source of original information, i.e., most probable cause leading to event $P_{27}$, through a network of collateral effects $\left\{P_{1},...,P_{26}\right\}$. The indicated root cause is confirmed to be correct by a series of automatically logged system events as well as service actions. Interestingly, the event log messages $P_{16}$ and $P_{23}$ follow the network’s direction of time i.e., from cause to effect (causal inference), while the logged service actions related to $P_{1}$ and $P_{23}$ follow the reversed time order, i.e., from effect to cause (diagnostic inference). Hence, the last logged service action $P_{1}$ appears as a direct effect of root cause $P_{0}$ in the network. This observation is promising, with regard to the automation of reliable data-driven diagnostics for technological complex systems.

## 4. Conclusions

To fully understand a complex system’s dynamical behavior, it is essential to identify its main sources of causal influence affecting downstream elements throughout the system. We empirically show that spectral centrality analysis of its causal network as approximated by standard transfer entropy allows one to accurately and consistently identify the most important node(s) of original information representing the most probable cause(s) or driver(s) of disturbance in the system. The ranking algorithm we use compares favorably against the alternative algorithm for multivariate information transfer estimation in causal analysis of two nonlinearly coupled Lorenz systems. In addition, it shows to be accurate, consistent and efficient, identifying the alternately driving and driven Lorenz subsystems, as well as the driving force within either subsystem, over time. Finally, the ranking algorithm correctly traces back the original disturbance within a high-dimensional technological complex system from sampled time series of several hundreds of parameters. Considering the high-dimensionality of observations across multiple time and spatial scales from such systems, we conclude that the inherent robustness of spectral centrality to semi-metricity of directed networks makes it a viable option for reliable and scalable diagnostics. Additionally, spectral centrality ranking allows for feature selection and is particularly useful in identifying long-term effects.

Regardless of the computational costs, state-of-the-art multivariate causal inference methods may be the better choice to account and control for unobserved variables or capture synergistic interactions. However, comprehensive comparison of our method with these approaches is beyond the scope of this study and therefore recommended for future research.

We thank David Sigtermans for our many inspiring discussions and Leonardo Barbini for his valuable feedback on the causal analysis results of the Lorenz systems. Finally, we are grateful to Simon Streicher for his implementation and helpful suggestions using it.

## Data Availability

The source code for simulation of time series representing coupled Lorenz systems, is available on https://osf.io, accessed on 19 March 2021.
